# Precision medicine and machine learning towards the prediction of the outcome of potential celiac disease

**DOI:** 10.1038/s41598-021-84951-x

**Published:** 2021-03-11

**Authors:** Francesco Piccialli, Francesco Calabrò, Danilo Crisci, Salvatore Cuomo, Edoardo Prezioso, Roberta Mandile, Riccardo Troncone, Luigi Greco, Renata Auricchio

**Affiliations:** 1 Department of Mathematics and Applications “Renato Caccioppoli”, University of Naples “Federico II”, Via Cintia, Monte S. Angelo, 80126 Naples, Italy; 2 Department of Translational Medical Sciences, University of Naples “Federico II”, Naples, Italy; 3 European Laboratory for the Investigation of Food Induced Diseases (ELFID), University of Naples “Federico II”, Naples, Italy

**Keywords:** Coeliac disease, Computer science, Scientific data

## Abstract

Potential Celiac Patients (PCD) bear the Celiac Disease (CD) genetic predisposition, a significant production of antihuman transglutaminase antibodies, but no morphological changes in the small bowel mucosa. A minority of patients (17%) showed clinical symptoms and need a gluten free diet at time of diagnosis, while the majority progress over several years (up to a decade) without any clinical problem neither a progression of the small intestine mucosal damage even when they continued to assume gluten in their diet. Recently we developed a traditional multivariate approach to predict the natural history, on the base of the information at enrolment (time 0) by a discriminant analysis model. Still, the traditional multivariate model requires stringent assumptions that may not be answered in the clinical setting. Starting from a follow-up dataset available for PCD, we propose the application of Machine Learning (ML) methodologies to extend the analysis on available clinical data and to detect most influent features predicting the outcome. These features, collected at time of diagnosis, should be capable to classify patients who will develop duodenal atrophy from those who will remain potential. Four ML methods were adopted to select features predictive of the outcome; the feature selection procedure was indeed capable to reduce the number of overall features from 85 to 19. ML methodologies (Random Forests, Extremely Randomized Trees, and Boosted Trees, Logistic Regression) were adopted, obtaining high values of accuracy: all report an accuracy above 75%. The specificity score was always more than 75% also, with two of the considered methods over 98%, while the best performance of sensitivity was 60%. The best model, optimized Boosted Trees, was able to classify PCD starting from the selected 19 features with an accuracy of 0.80, sensitivity of 0.58 and specificity of 0.84. Finally, with this work, we are able to categorize PCD patients that can more likely develop overt CD using ML. ML techniques appear to be an innovative approach to predict the outcome of PCD, since they provide a step forward in the direction of precision medicine aimed to customize healthcare, medical therapies, decisions, and practices tailoring the clinical management of PCD children.

## Introduction

Potential Celiac patients are characterized by genetic predisposition to celiac disease (CD), presence of CD specific antibodies (anti-human tissue transglutaminase antibodies and anti-endomysium) in the serum, but no morphological changes in the small bowel mucosa^1–7^. Only a small percentage of them showed significant clinical symptoms (and are started on a gluten free diet at time of diagnosis), while the majority progressed over several years (up to a decade) without any clinical problem or a progression of the small intestinal mucosal damage even if they continued a gluten containing diet, on long term follow up one third of them progressed to a clear pattern of CD mucosal damage. The real issue was to attempt to predict, at enrolment, who was more likely to progress to villous atrophy disease in order to prevent clinical and histological damage related to the disease. In a previous paper, we developed a traditional multivariate approach to predict, on the base of the information at enrolment (time 0), subjects more likely to develop the full-blown disease. Overall, a discriminant analysis model allowed to correctly classify, at entry, 80% of the children who would not develop a flat mucosa over follow-up, whereas approximately 69% of those who did develop flat mucosa are correctly classified by the starting parameters^1^.

As discussed by Wasserstein et al. in^8^, making conclusions based uniquely on linear models can give unhelpful information when clinical data are used. Among others, some of the well-known limitations of the linear models are: assumption about the distribution of the variables not controlled; non independency of the variables selected in the model; the models obtained, being hypothesis driven, may not respect the uncertainty about the biological significance of the variable selected; relative weakness of sample size leading to very large confidence intervals on follow up.

In this second phase, we adopted a machine learning approach to validate an innovative method to predict the outcome.

ML techniques were proposed to support clinical decision in studies where multiple features can affect outcomes. Recently, several studies produced seminal papers that invite the community to use such methods^9–19^. Obermeyer, Rajkomar et al.^9-11^ reviewed Artificial Intelligence methods currently used in medicine, while the impossibility to use large amount of data without an automatic code was discussed by Schwalbe and Wahl^12^. Also, the description of the “The All of Us Research Program” in^13^ and the recent review on deep learning by Piccialli et al.^14^ focused on these issues. The editorial office of The Lancet Respiratory Medicine^15^ gave some guidelines for ML, as done by other authors^16–18^. Beam et al.^19^ focused on guidelines for reproducibility of results. What was noticed in such studies was that ML is a powerful set of tools that help the extraction of significant features for the prediction of outcomes. Nevertheless, because of its wide range applicability, considerable caution in the interpretation of models was required to produce an innovative approach to clinical data. Common pitfalls and roadmap for the application of ML methods in the medical domain were deeply reviewed^20–23^.

Many studies adopted ML techniques effectively in various clinical frameworks for the prediction of outcomes. The main domain, where ML and other Data Analysis techniques are widely used, is cancer research and rheumatology, as pointed out respectively in the review by Hinkson et al.^24^ and Radstake et al. in^25^: in this case, also images are used to enrich the available data set. Images are also used in the detection of CD with the use of ML in^26,27^.

What is the most important feature—and maybe also the main drawback—is that the application of the ML techniques is model-free, data-driven, and intrinsically non-linear. ML takes advantage of all the available data, uses the different features known in the learning process: for example, the fields with categorical values can be converted into different numeric fields so that they are treated separately, without the need of ordering.

Our data set presents a temporal pattern due to the follow-up, with an increasing sparsity of the data as the follow-up is increased. For this reason, a ML strategy which considers the features collected at the enrolment allow to obtain a not increasing confidence interval for the final prediction, despite the decreasing sample size as the number of follow-ups increases. This provides a robust methodology, compared to the usual statistical parameters estimates.

In this study, we used ML for feature selection and for classification in a new condition such as CD and its multifactorial pathogenetic elements. Feature selection gives indications on the best predictive items in the dataset, while the classification result is given via threshold: it will give 1 (high risk) if the model output for a given value exceeds 50%, 0 otherwise.

Aims of this work was, starting from a follow-up dataset available for PCD, to apply Machine Learning (ML) to select most influent features and introduce predictive models to distinguish patients who developed duodenal atrophy from those who remained potential on a gluten containing diet.

## Materials and methods

### Prospective cohort features

A prospective cohort of potential celiac disease children (340) was followed up from diagnosis till maximum of 12 years^1^. Diagnosis was confirmed when children showed at least 2 positive anti-transglutaminase IgA and anti-endomysium serological tests and all duodenal biopsies performed (1 from the bulb and 4 from distal part of the duodenum) were not atrophic, according to Marsh-Oberhuber classification. All patients enrolled were also HLA DQ2 and/or DQ8 positive. Symptomatic children started a gluten-free diet at time of diagnosis. The others (280/340) continued a gluten containing diet and had clinical and serological evaluation every 6 months and histological examination every 2 years^1^. 42/280 (15%) developed a flat duodenal mucosa during follow-up, while 89/280 (32%) became completely seronegative and 149 remained potential during follow-up. Risk factors associated with development of villous atrophy were investigated by log-rank test to compare the effect of factors on survival and a multivariate analysis was used to deal with the correlations among the variables considered.

The study was carried out according to the Helsinki II Declaration and was approved by the Ethical Committee of the School of Medicine of the University of Naples Federico II, Protocol n. 191/06. The present research involving human participants under the age of 18 years (including donors of tissue samples). Each parent (and/or legal guardian) gave a fully informed consent to the participation of their child to the study and to the use of their biological samples for research purpose. The form is available on request at 'r.auricchio@unina.it'.

The datasets analysed during the current study are available from the corresponding author on reasonable request.

### Data cleaning and preprocessing

**Figure 1:**
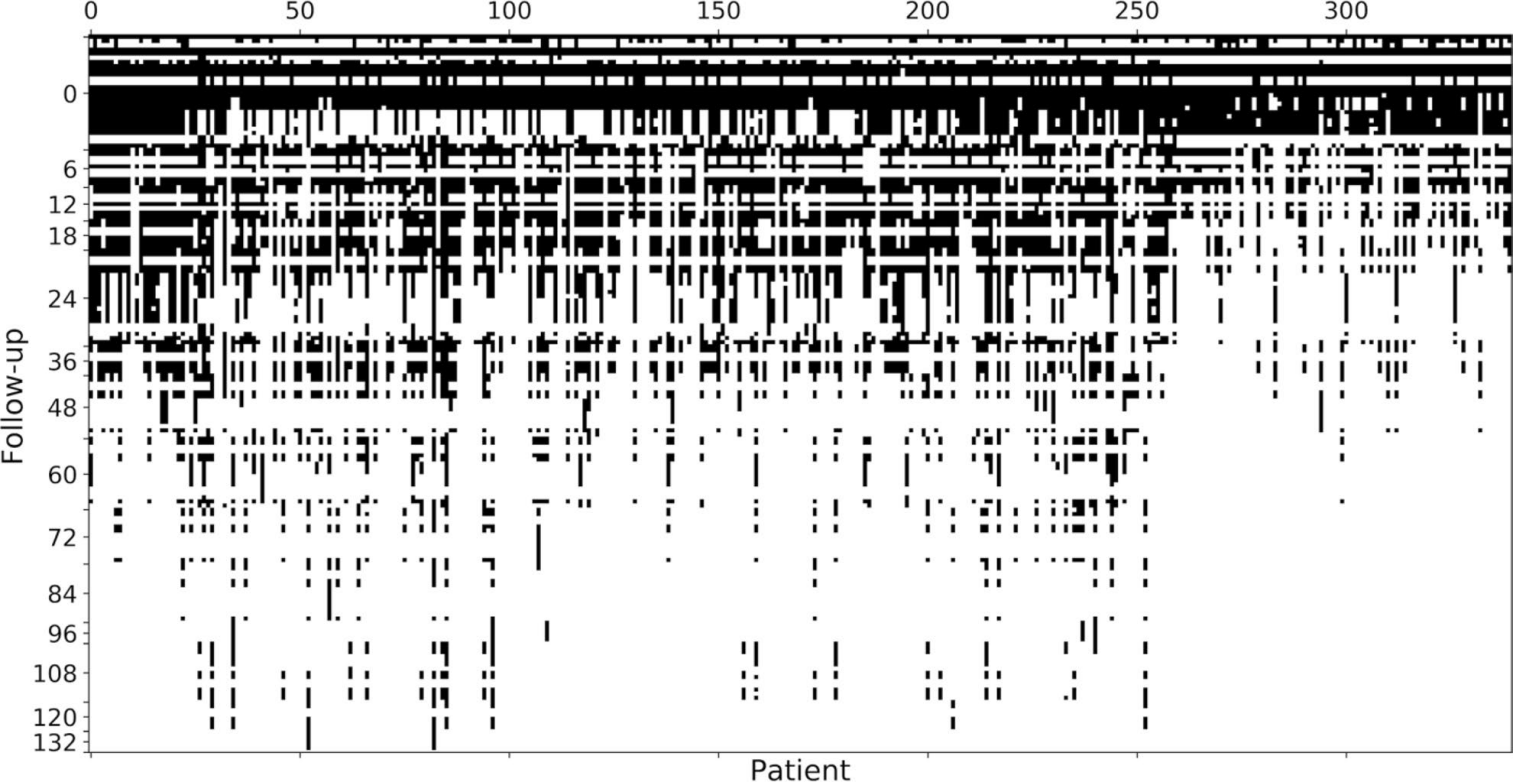
A spy plot about the presence of values on the whole dataset: black indicates available data, white missing. The numbers on the ticks on the vertical axis indicates the follow-up months, while the ticks without numbers indicate the gap between two separate follow-ups. The pattern is typical of longitudinal studies: such distribution highlights the problem when results are produced via the classical descriptive statistical approach, where the model is confirmed in terms of confidence intervals and probability distributions with a quite limited data set. ML with tree techniques can overcome this feature using all the available data.

**Figure 2:**
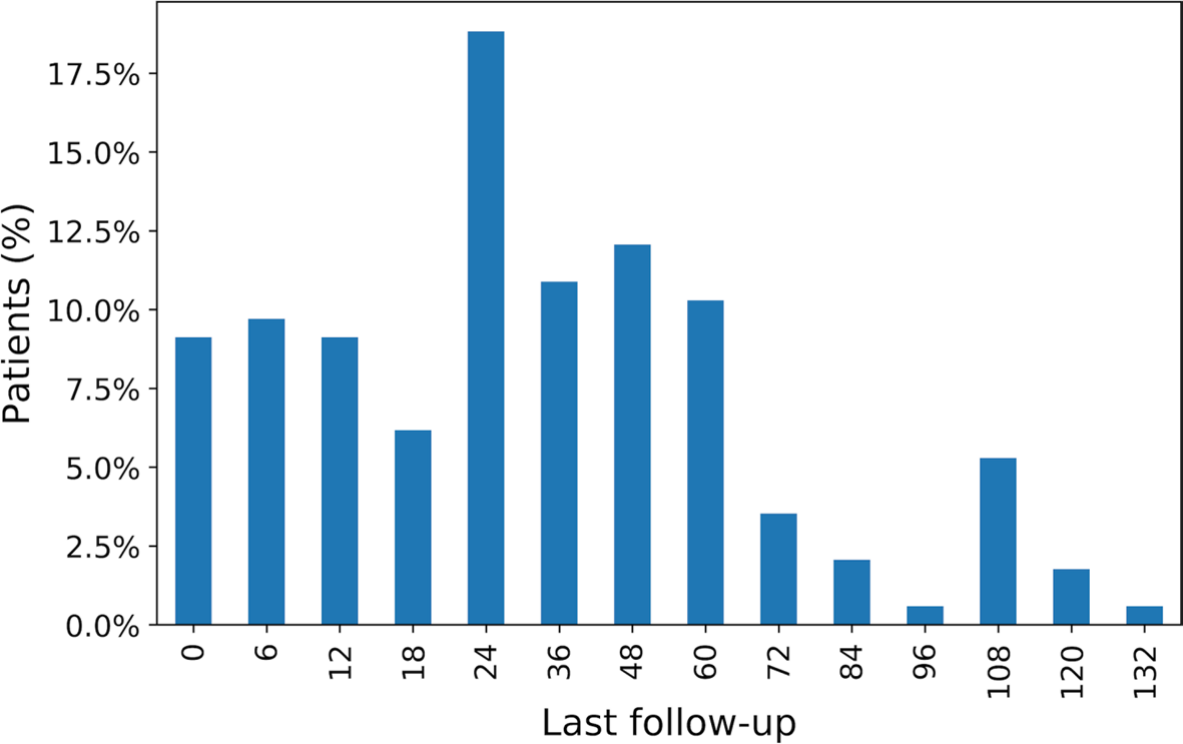
The percentage of patients with last follow-up. Different motivations can cause the interruption of follow-up, such as the onset of the disease, familial and logistic problems, mobility, unavailable blood sample.

Starting from the available dataset, a data cleaning and pre-processing step was required. The analysed dataset contains both categorical and numerical features. Some of them present missing data values, however these features have still been considered either in feature reduction and classification tasks. In this context, results are mainly affected by the poor filling of the data and the imbalance of the predicted targets. Both issues can be easily explained: for the first one, the follow ups are available at different time lengths according to different individuals (censored data); for the second issue, the diagnosis of the overt CD happens in about 30% of the cases, while ML works better if the outcomes are balanced. In Figs. 1 and 2 we reported disposable data for each time point of the follow-up and the distribution of the patients by the available follow-up. In this work, the results of the clinical tests in the successive follow ups were not considered for two main reasons: first of all, the objective of this work is to make a prediction of the outcome at the time of the diagnosis of PCD; furthermore, there were not enough patients whose sequence of clinical results in the consecutive follow ups is consistently present.

The fields with categorical values were converted into multiple numeric fields through one-hot encoding^28^; indeed, it would make no sense to apply ordinal encoding to the ordinal categories, because they may contain missing data, and therefore there could be a loss of significance if the missing data would be replaced with a numeric value. Finally, the considered features were those at the first follow up (at time 0).

Then, ML was applied to the dataset with two aims: feature selection and supervised classification for the villous atrophy development prediction. In both cases, ML methodologies are characterized in general by a model, whose parameters are adjusted by inferring on a subset of the dataset called *training set*, then the goodness of such model is evaluated by computing a score on a disjoint set of instances called *test set*. Each model also depends on non-trainable parameters called *hyperparameters* that influence the prediction; it is crucial to find good settings for them, and this operation is called hyperparameter tuning. Regarding this point, classical approaches consist in evaluating a model with different hyperparameters in a subset of the dataset, disjoint from both the training set and the test set, called *validation set*. Because of too few available instances, the procedure of splitting the dataset into train, validation and test sets is not recommended, because there is a risk of losing the statistical representativity of the training set. In order to alleviate this problem, a k-fold cross validation approach has been used, with k = 10. More in detail, the dataset was partitioned in 10 disjoint subsets. At each iteration of the cross validation, 9 subsets become the training set, while the remaining subset is chosen as the validation set. Since the number of occurrences of the diagnosed CD was 30% of the total number of samples, a more suitable version of the tenfold cross validation, called *stratified cross validation*, was deployed. This guarantees the same percentage of the distribution of the CD targeted instances in each of the 10 subsets. Then, for each choice of hyperparameters on a given model, the average of the validation scores obtained on all the iterations of the cross validation is computed, then, finally, the best hyperparameter configuration is chosen by taking the configuration corresponding to the best value between such scores. The justification of using this approach is that the classical split into train, validation and test sets presupposes the representativity of the entire dataset being preserved in each subset. Unfortunately, this assumption cannot be made for our data set, given the small number of PCD patients. Therefore, the same dataset has been used in both feature selection and classification, and the hyperparameter tuning has been validated by using the discussed k-fold stratified cross validation. This is a general strategy which limits the overfitting phenomena^29,30^.

## The proposed ML workflow

**Figure 3:**
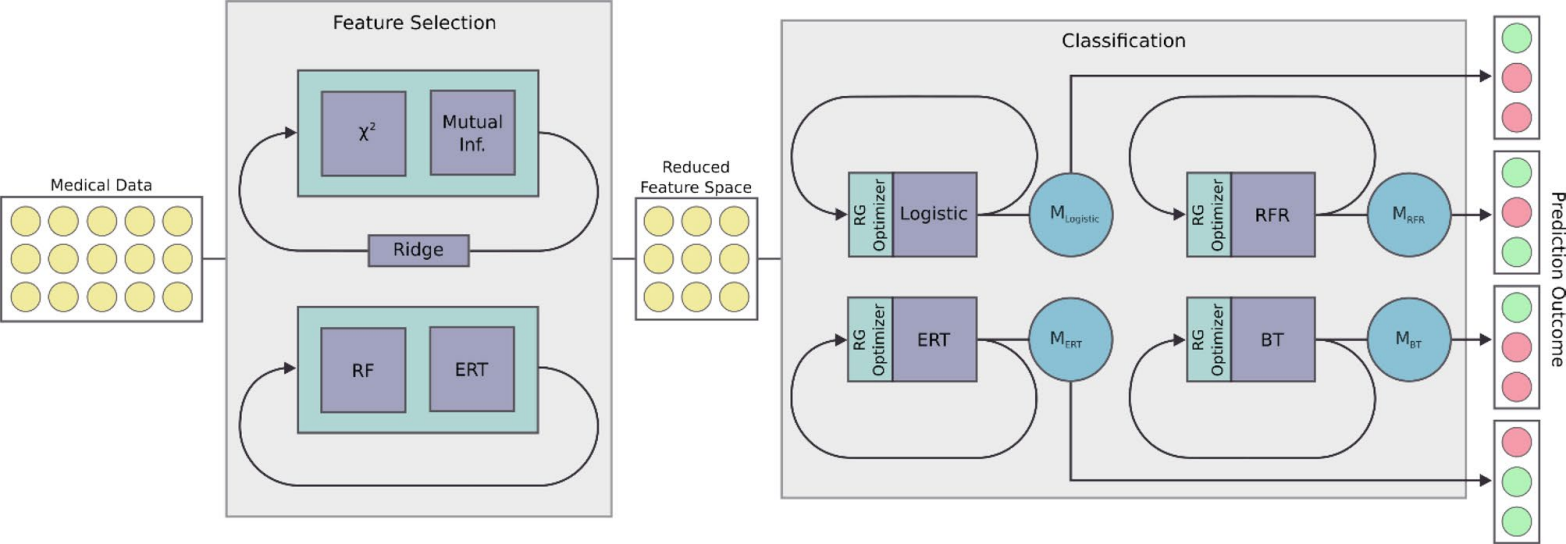
The proposed ML workflow for outcome prediction. The medical data are reduced in terms of features by a Feature Selection scheme (left grey box), then the predicted outcome is obtained by using ML models in a Classification procedure (right grey box). For abbreviations, see “ML models” section.

In Fig. 3 we present a ML workflow which briefly summarizes the computational procedure for the PCD children categorization. The medical data are processed with a Feature Selection scheme (left gray block), then in the obtained reduced feature space, a Classification phase (right gray block) is used to the outcome predictions. In this section, we recall the ML background about the models, the feature selection and the classification^28^.

### ML models

For the Feature Selection scheme, the following models have been considered: (i) univariate analysis via chi squared and mutual information statistical tests, with validation through the Ridge classifier, (ii) Random Forests (RF) and Extremely Randomized Trees (ERT). For the Classification phase, we consider: (i) RF, (ii) ERT, (iii) Boosted Trees (BT), (iv) Logistic regression (LR). Details of the ML models are here reported:

- The Ridge classifier, used in the statistical tests, is a linear model whose coefficients are obtained by solving the mean squared error optimization problem with a quadratic penalization term on the coefficients multiplied by a hyperparameter balancing the regularization.
- RF is a methodology relying on averaging random versions of decision tree models to reduce the inherently high variance from each tree model. The construction of each tree is done by satisfying properties related to discrimination criteria on each tree node. For the classification, the predicted result is obtained by a majority vote on the statement of each decision tree and for feature selection, the importance of the model feature is determined by the percentage of the features that are present in the decision tree nodes.
- ERT is based on the ensemble of more randomly built decision trees than RF, allowing less variance, paying a greater bias. The weight of the important features is assessed as described for RF.
- BT uses the idea to fit a sequence of simple decision trees with an assigned rule. Given an underlying function that maps the *feature space* in the *target space*, the boosting procedure approximates it through an *additive weighted expansions* technique; this procedure produces a good fit of the predicted values. In order to reduce the phenomenon of overfitting, a combined bagging-boosting procedure for the least-squares fitting of additive expansions is adopted.
- LR is a linear model which is used to predict the outcome in a probabilistic way. More in detail, the probability distribution of the predicted outcome is modelled by a logistic function. In this work, we adopted a modified version of LR where several penalization strategies are implemented, allowing to improve the training of the model through the optimization.

### Feature selection scheme

We detail the feature selection block in Fig. 3. Given a data set, the objective is to extrapolate a subset of the features which are most representative. This methodology has a double significance: in the context of clinical diagnosis, it allows to detect risk factors; in the context of ML, it is a way to alleviate the problem of the curse of dimensionality, where the dimensionality of the features is numerically comparable to the dimensionality of the samples. Since the number of samples is relatively low, a procedure of cross validation has been used to validate the choice of the features. More in detail, given the 10 splits of the cross validation, at each step the feature importance of the trained model is computed; then, for each model, the average of the feature importance in the whole cross validation procedure is considered. Finally, we reordered the features according to the sum of the feature importances from the four considered models. In conclusion, the reduced feature space is obtained so that the cumulative importance value (CIV), with respect to the overall sum, reached the value of 75%.

This approach can be justified as follows. Since a single Feature selection model is not able to extract the whole set of complex relationships between our data, an ensemble of the four methods is considered to enhance the generalization of the best feature detection process. Furthermore, the CIV criteria are used because it has the advantage of not choosing aprioristically the cardinality of the best features set, but rather to adaptively determine it depending on the magnitude of the feature importance obtained.

### Classification phase

The reduced feature space is processed in a classification phase. Indeed, the attempt to classify, at enrolment (time 0), who was more likely to progress to villous atrophy can be treated as a binary classification problem. For each ML model Λ in Fig. 3 (with Λ = Logistic, RFR, ERT, BT), an optimization through the hyperparameter tuning is done, as previously described, in order to generate the optimized model M_Λ_. M_Λ_ that has been used for the prediction of the final outcome. The results of the classification were validated through a tenfold cross validation.

The models that have been considered for this type of problem are those based on tree methodologies: Random Forest, Extremely Randomized Trees, and Boosted Trees. The Boosted Trees method, unlike the other two, is based on the progressive training of trees in a sequential way, i.e. a tree was trained starting from the previously trained tree through gradient boosting. This approach was considered to confirm that selected features have powerful predicting efforts.

In this work, the classification problem can hardly be seen as a regression problem, since the label had only two values. Linear logistic regression was used in this report. The goal was to find linear coefficients such that the logistic distribution obtained from the linear combination of the features with these coefficients can approximate the output to a correct prediction.

## Results

**Figure 4:**
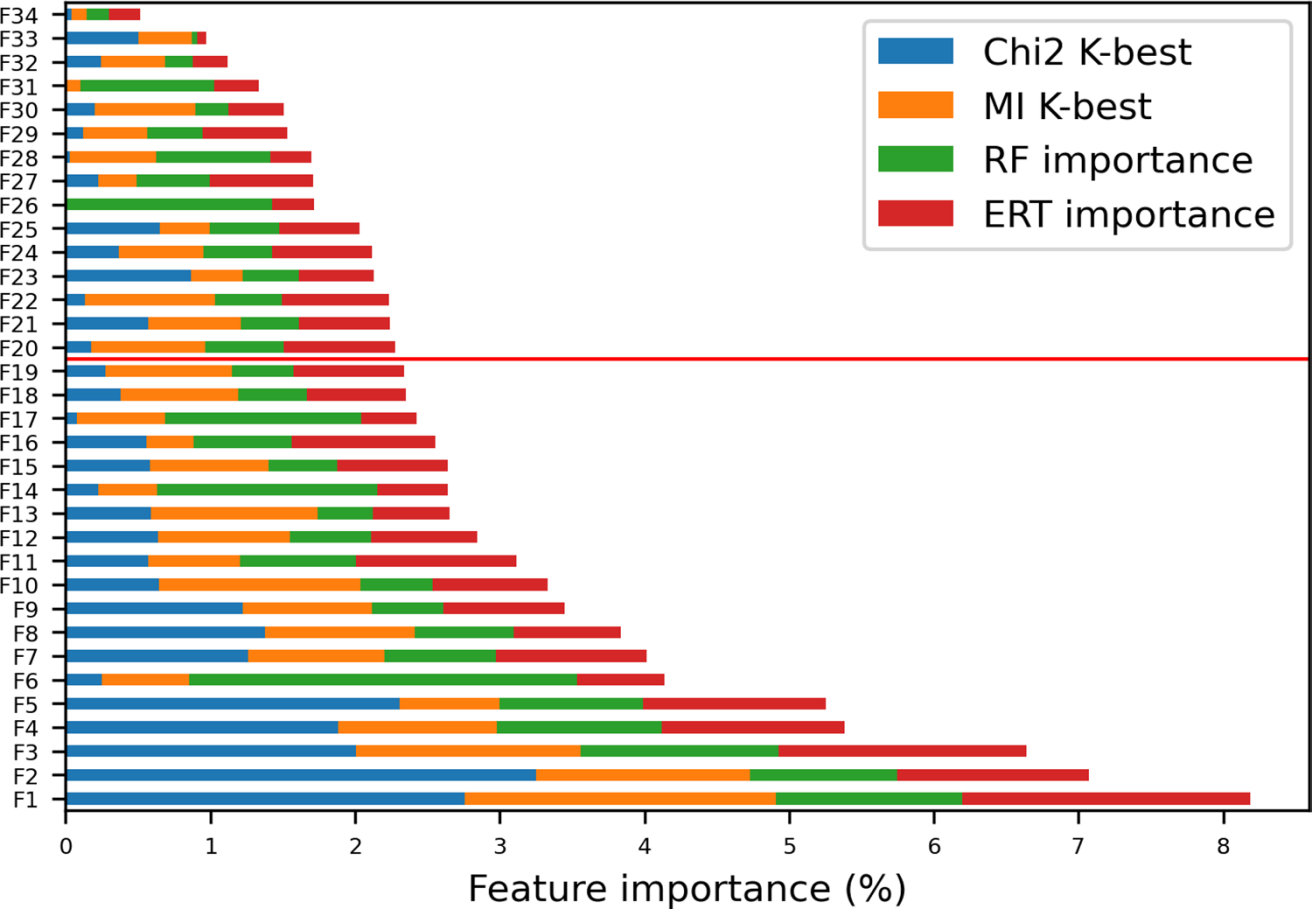
Cumulative feature importance. This graph was obtained by normalizing each feature relevance value by selecting a model for overall relevance and then by sorting the normalized relevance on the considered models. By using this methodology, the best selected features were chosen so that the Cumulative Importance Value (CIV), concerning the overall sum, reaches the value of 75%: the red line divides the selected features from the others. For the description of the features, see Table 1.

As far as the feature selection is concerned, univariate analysis allowed to select only the features that satisfy hypothetical statistical tests, hence chi-square tests and mutual information were chosen. The most relevant features were obtained by a grid search strategy by ranking the number K of the selected features from 10 to 30. The adopted grid search criterion was the maximum of the cross-validated AUC score by training a Ridge classifier with the regularization strength equal to 0.01. The optimal value for the number of features was found to be 15 for the Chi-square test and 19 for the mutual information test. Results obtained for the top 34 features are reported in Fig. 4, while the description of such features is summarized in Table 1. Figure 4 shows the values via the cumulative weight of each variable giving the relevance of features, where the one hot encoding is applied to categorical ones.

**Table 1:** Feature description.

Feature	Type	Description
F1	Categorical	Age group at diagnosis (grouped in below 3/between 3 and 10/ over 10): between 3 and 10 years
F2	Categorical	Anti-tTG2 IgA deposit in duodenal mucosa at time of diagnosis: low positivity
F3	Categorical	HLA haplotype: DQ2/DR7
F4	Categorical	IL2/IL21 haplotype: GG
F5	Categorical	Anti-tTG2 IgA deposit in duodenal mucosa at time of diagnosis (grouped in present/absent/weak): weak
F6	Categorical	Age at first biopsy/diagnosis (grouped by integer age)
F7	Categorical	IL12 haplotype: TT
F8	Categorical	Height of villi in the first biopsy (grouped in normal/pathological/variable): variable
F9	Categorical	SH2B3 haplotype: TT
F10	Categorical	CCR haplotype: TC
F11	Categorical	Intra-epithelial lymphocytes in first biopsy < 34 cells/mm^2^
F12	Categorical	RGS1 haplotype: AC
F13	Categorical	Anti-endomysium antibodies at the first biopsy (grouped in absent/present/weak/very weak/patchy): weak
F14	Numerical	Gamma delta intra-epithelial infiltration in first biopsy
F15	Categorical	OLIG3 haplotype: AG
F16	Categorical	Villi/crypt ratio in first biopsy (grouped in normal/pathologic): normal
F17	Categorical	Thyroiditis in family
F18	Categorical	LPP haplotype: AC
F19	Categorical	Inflammatory infiltration in the lamina propria (grouped in present/absent): present
F20	Categorical	Depth of crypts in first biopsy (grouped in normal/pathologic): normal
F21	Numerical	CD3 in crypts
F22	Categorical	IL18RAP haplotype: TT
F23	Categorical	TAGAP haplotype: TC
F24	Categorical	REL haplotype: AA
F25	Categorical	Marsh in first biopsy (grouped in M0/M1/M3): M1
F26	Numerical	Anti-tTG2 value compared to the upper limit of the normal
F27	Categorical	Celiac disease in family
F28	Categorical	Villi/crypts ratio (grouped in normal/pathological): normal non è categorical?
F29	Categorical	Sex: male
F30	Categorical	SCHIP1 haplotype: AA
F31	Numerical	CD25 infiltration in the lamina propria in the first biopsy
F32	Categorical	Vitiligo in family
F33	Categorical	Hypercholester in family
F34	Categorical	Diabetes in family

Features are numbered in order of relevance, as obtained by feature selection and reported starting from the most important feature. The first 19 are the one selected for the classification process, a red line has been added to divide the selected features among the others. It can be noticed that the selected features include mainly features of the child (age, age at biopsy), his genetic profile and data related to the infiltration of the Small Intestinal Mucosa (including mucosal production of anti-tTG antibodies).

**Figure 5:**
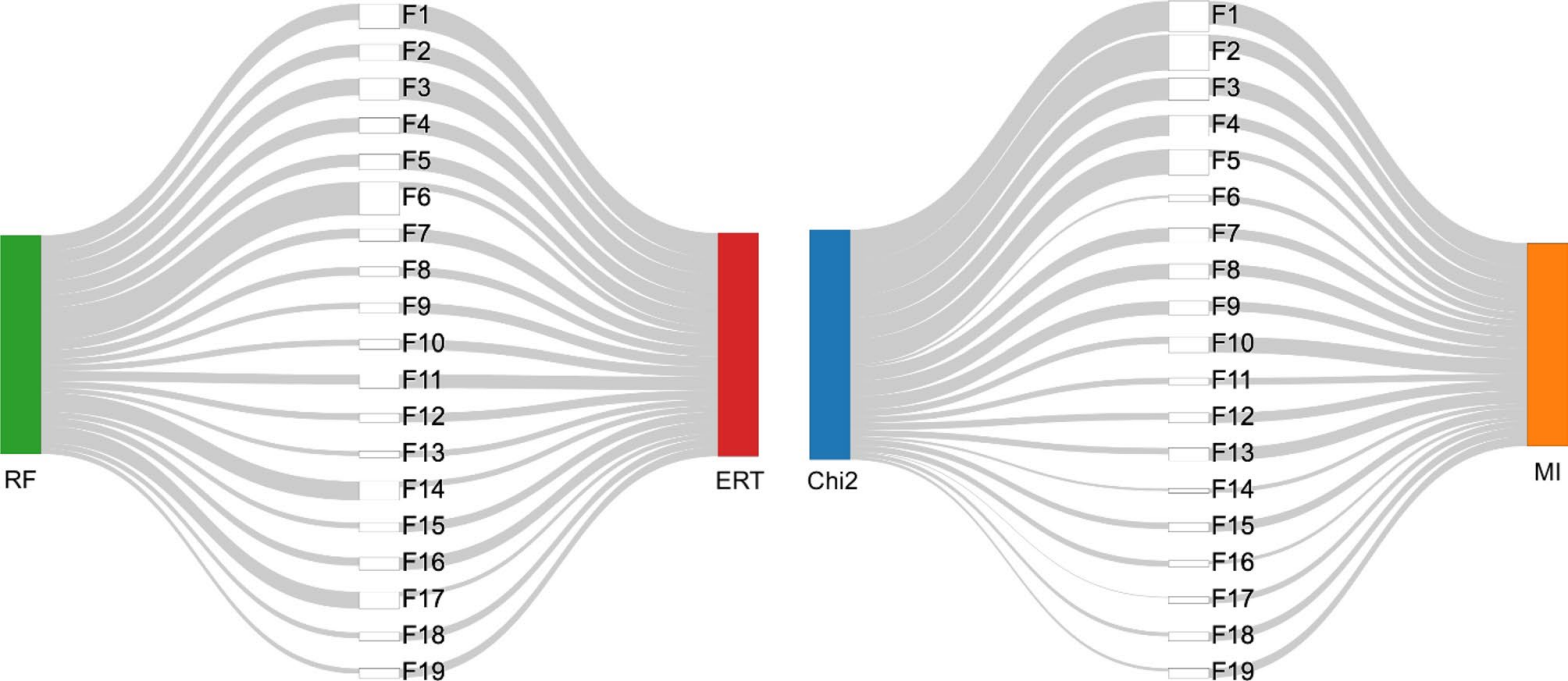
Feature contribution to the four models. In this graph, the thickness of the grey lines indicates how much the feature impacts the results obtained by the model. For legend on the models, see Fig. 3, for the description of the features, see Table 1.

According to the threshold set to 75% of CIV, 19 features were chosen, denoted by F1–F19 (see Table 1). In Fig. 5 the contribution of the selected features to the four models is shown.

It can be noted that the selected features include mainly features of the child (age, age at biopsy), his genetic profile and data related to the infiltration of the Small Intestinal Mucosa (including mucosal production of anti-tTG2 antibodies) at time 0.

The validation of the model was given in terms of accuracy, sensitivity, specificity. Then, the Area Under the Curve (AUC) and the Receiver Operating Characteristics (ROC) were computed to estimate the performance of the proposed methodology. The results of classification are reported in the form *mean* ± *standard deviation* of the 10 cross validations in Table 2. All the methods report an accuracy above 75%, but there are deep differences when other parameters are considered. Both Random Forest (RF) and Extremely Randomized Trees (ERT) have the highest scores in terms of accuracy and specificity, but because of the low specificity score, they do not perform sufficiently for predicting true positives. Instead, both Boosted Trees (BT) and Logistic Regression (LR) provide the best scores for specificity without a great loss in terms of True Negative cases. In particular, we observe that BT has a higher value of accuracy, specificity and AUC than LR, at the small cost in terms of sensitivity, but since its standard deviation is smaller than LR, BT can be considered as the best model. Building sequential decision trees through bagging-boosting techniques has been proven helpful for this task. Furthermore, if we consider the ROC AUC score, the two aforementioned methods reported the best results, showing that such models have a good predictive power, even though the data set is unbalanced. As expected, all the optimized models report the highest ROC AUC value than the non-optimized values (except for the ERT). The results related to this application showed that there are some Supervised Learning Models, like in this case BT an LR, which can detect patterns which were peculiar only to the relatively few cases that develop CD.

**Table 2:** Classification results.

Model	Accuracy (TP + TN)/N	Sensitivity TP/(TP + FN)	Specificity TN/(TN + FP)	ROC AUC
RF	0.84 ± 0.04	0.06 ± 0.12	**0.98 ± 0.03**	0.52 ± 0.06
RF (optimized)	0.83 ± 0.05	0.22 ± 0.21	0.94 ± 0.05	0.58 ± 0.11
ERT	**0.86 ± 0.03**	0.16 ± 0.18	**0.98 ± 0.03**	0.57 ± 0.09
ERT (optimized)	0.85 ± 0.03	0.06 ± 0.12	**0.98 ± 0.02**	0.52 ± 0.05
BT	0.81 ± 0.07	0.38 ± 0.17	0.89 ± 0.07	0.63 ± 0.10
BT (optimized)	0.80 ± 0.08	0.58 ± 0.18	0.84 ± 0.08	**0.71 ± 0.09**
LR	0.77 ± 0.07	0.58 ± 0.20	0.80 ± 0.08	0.67 ± 0.10
LR (optimized)	0.75 ± 0.08	**0.60 ± 0.24**	0.78 ± 0.10	0.69 ± 0.11

TP = true positive, TN = true negative, FP = false positive and FN = false negative. The results of classification are reported in the form *mean* ± *standard deviation* of the parameters obtained by 10 cross validations. All the methods report an accuracy above 75%. Specificity also is always above 75%, with two of the considered methods over the 98%, while the best performance in terms of sensitivity is 60%. Then, the Area Under the Curve and the Receiver Operating Characteristics are reported to estimate the performance in terms of accurate classification of the proposed methodology. For all results, the best-obtained scores are highlighted in bold. For the ROC AUC score, two methods (BT & LR) report better results, showing that such models have a good predictive power even though the data set is unbalanced. As expected, all the optimized models report the highest ROC AUC value than the non-optimized values (except for the ERT), due to the hyperparameter tuning.

The trained model can be used for future classification of PCD starting from the clinical data, giving an indication to the paediatrician in the domain of precise medicine.

### Comparison with previous work on PCD

In the previous paper^1^ a stepwise discriminant analysis was used to select variables able to differentiate children who became celiac from those who remained potential over 8 years follow up. A Discriminant score (D-Score) was calculated by multiplying the normalized value of each variable included in the stepwise discriminant equation to its respective regression coefficient. From the score, the individual probability to be assigned to one or the other group was derived: we classified (predicted) the individuals into CD or not-CD group, using the selected variables, blinded to the final diagnosis.

In this work we are able to categorize PCD that can more likely develop CD using ML. Starting from the available dataset, the models are trained by the items and can be used for the outcome prediction. This overcomes the previously available linear model and proposes a novel classification of PCD based on ML.

## Discussion

ML methods showed that some clinical and laboratory features have an important predictive power to forecast the development of villous atrophy. We wish to highlight this statement in order to guide the reader that could be sceptical about automatic indicators: what we found, is that the features selected by ML are roughly the same that give the important information in the linear model available in^1^, but the prediction that ML offers is significantly more accurate when compared to previous methods.

The issue of this domain of research is to support the clinical decision making for the management of potential celiac children at entry, based on informations/variables available at the first clinical and laboratory work up. Indeed, the majority of potential CD do not develop a full-blown disease within 8 years of follow up. Up to one third decrease the production of their main feature: the anti-human transglutaminase antibodies (Anti tTG2). On the other end, for the 30–35% who eventually develop villous atrophy over follow-up, an accurate prediction at time 0 (diagnosis) might prevent the progressive pathological process leading to a full small bowel mucosal destruction. We previously used a traditional multivariate approach to estimate, by a discriminant model, at diagnosis, which individual is more likely to develop villous atrophy over time, reaching an accuracy of prediction close to 70%. But the multivariate approach requires assumptions about the quality of variables used to develop the model which might be not fully appropriate to many clinical data. The independency of each variable by the other variables, which is a requirement for the best multivariate model is rarely respected: there is, at the end, at least a 30% misclassification. Alternatively, a hypothesis free method does not require a specific distribution of each variable neither it requires mutual independency of the variables. It may be finally simpler to fit the clinical judgement of the physician. For example, our trained BT (optimized) model can categorize PCD starting from the 19 selected features with an accuracy of 0.80, sensitivity of 0.58 and specificity 0.84.

This gives a clear indication to physician on the relevance of collecting data on genetic profile and infiltration of the Small Intestinal Mucosa for PCD and raises the question about the opportunity to put a child on gluten-free diet starting from these features before the development of the full blown CD. ML indications can move towards precision medicine also the detection of CD, as done in other diseases with similar workflows, as shown for the evaluation of cardiometabolic risk and risk of developing diabetes^2–7,31–34^.

Celiac Disease automated diagnosis is not new to computer-assisted systems, which have been explored since 2008^35^; spatial domain, transform domain, scale-invariant and and spatio-temporal features have been applied to several aspects of CD diagnosis, especially to the subjective interpretation of the intestine small mucosal immaginery^36^. But artificial intelligence, machine learning and deep learning do require large amount of data, in order to produce reliable results, and this is often one of the major caveat of clinical studies.

This work also presents some limitations. The relatively low number of data samples, with the outcome being unbalanced, and the lack of test samples from an external cohort are critical issues. It is indeed known that ML applied to small, sparse and heterogeneous data is challenging in terms of model contextualization, validation procedure and the classification accuracy. Moreover, about the limitations of the proposed ML workflow, we are working on a semi-automatic strategy of hyperparameter tuning in both the feature selection scheme and the classification phase, since not all the possible combinations of hyperparameters have been tested. Strategies, like the usage of the cross validation for the choice of the best hyperparameters in both feature selection and classification, should allow improving the model performance.

## Data Availability

The study was carried out according to the Helsinki II Declaration and was approved by the Ethical Committee of the School of Medicine of the University of Naples Federico II, Protocol n. 191/06. The present research involving human participants under the age of 18 years (including donors of tissue samples). Each parent (and/or legal guardian) gave a fully informed consent to the participation of their child to the study and to the use of their biological samples for research purpose. The form is available on request at 'r.auricchio@unina.it'. The datasets analysed during the current study are available from the corresponding author on reasonable request.

## Abbreviations

PCD: Potential celiac patients
CD: Celiac disease
ML: Machine learning
AUC: Area under the curve
ROC: Receiver operating characteristics

## References

[1] Auricchio R, Mandile R, Del Vecchio MR (2019). Progression of celiac disease in children with antibodies against tissue transglutaminase and normal duodenal architecture. Gastroenterology.

[2] Auricchio R, Tosco A, Piccolo E, Galatola M, Izzo V, Maglio M, Paparo F, Troncone R, Greco L (2014). Potential celiac children: 9-year follow-up on a gluten-containing diet. Am. J. Gastroenterol..

[3] Volta U, Caio G, Giancola F, Rhoden KJ, Ruggeri E, Boschetti E, Stanghellini V, De Giorgio R (2016). Features and progression of potential celiac disease in adults. Clin. Gastroenterol. Hepatol..

[4] Trovato CM, Montuori M, Valitutti F, Leter B, Cucchiara S, Oliva S (2019). The challenge of treatment in potential celiac disease. Gastroenterol Res Pract..

[5] Noh J, Yoo KD, Bae W (2020). Prediction of the mortality risk in peritoneal dialysis patients using machine learning models: A nation-wide prospective cohort in Korea. Sci. Rep..

[6] Heo J, Park SJ, Kang SH, Oh CW, Bang JS, Kim T (2020). Prediction of intracranial aneurysm risk using machine learning. Sci. Rep..

[7] Rawshani A, Eliasson B, Rawshani A (2020). Adipose tissue morphology, imaging and metabolomics predicting cardiometabolic risk and family history of type 2 diabetes in non-obese men. Sci. Rep..

[8] Wasserstein RL, Schirm AL, Lazar NA (2019). Moving to a world beyond “p< 0.05”. Am. Stat..

[9] Obermeyer Z, Emanuel EJ (2016). Predicting the future—big data, machine learning, and clinical medicine. N. Engl. J. Med..

[10] Obermeyer Z, Lee TH (2017). Lost in thought—the limits of the human mind and the future of medicine. N. Engl. J. Med..

[11] Rajkomar A, Dean J, Kohane I (2019). Machine learning in medicine. N. Engl. J. Med..

[12] Schwalbe N, Wahl B (2020). Artificial intelligence and the future of global health. Lancet.

[13] The All of Us Research Program Investigators (2019). The “All of Us” research program. N. Engl. J. Med..

[14] Piccialli, F., Di Somma, V., Giampaolo, F., Cuomo, S., & Fortino, G. A survey on deep learning in medicine: Why, how and when?. Information Fusion. ISO 690 (2020).

[15] Medicine TLR (2018). Opening the black box of machine learning. Lancet Respir Med..

[16] Peterson ED (2019). machine learning, predictive analytics, and clinical practice: Can the past inform the present?. JAMA.

[17] Shah NH, Milstein A, Bagley SC (2019). Making machine learning models clinically useful. JAMA.

[18] Pencina MJ, Goldstein BA, D'Agostino RB (2020). Prediction models—development, evaluation, and clinical application. N. Engl. J. Med..

[19] Beam AL, Manrai AK, Ghassemi M (2020). Challenges to the reproducibility of machine learning models in health care. JAMA.

[20] Salzberg SL (1997). On comparing classifiers: Pitfalls to avoid and a recommended approach. Data Min. Knowl. Disc..

[21] Riley P (2019). Three pitfalls to avoid in machine learning. Nature.

[22] Liu Y, Chen PC, Krause J, Peng L (2019). How to read articles that use machine learning: Users' guides to the medical literature. JAMA.

[23] Doshi-Velez F, Perlis RH (2019). Evaluating machine learning articles. JAMA.

[24] Hinkson IV, Davidsen TM, Klemm JD, Chandramouliswaran I, Kerlavage AR, Kibbe WA (2017). A comprehensive infrastructure for big data in cancer research: Accelerating cancer research and precision medicine. Front. Cell Dev. Biol..

[25] Pandit A, Radstake TRDJ (2020). Machine learning in rheumatology approaches the clinic. Nat. Rev. Rheumatol..

[26] Hujoel IA, Murphree DH, Van Dyke CT, Choung RS, Sharma A, Murray JA, Rubio-Tapia A (2018). Machine learning in detection of undiagnosed celiac disease. Clin. Gastroenterol. Hepatol..

[27] Molder A, Balaban DV, Jinga M, Molder C-C (2020). Current evidence on computer-aided diagnosis of celiac disease: Systematic review. Front. Pharmacol..

[28] Friedman, J., Hastie, T. & Tibshirani, R. *The Elements of Statistical Learning,* Vol. 1. No. 10 (Springer Series in Statistics, 2001).

[29] Varma S, Simon R (2006). Bias in error estimation when using cross-validation for model selection. BMC Bioinform..

[30] Duda, R.O., Hart, P.E. & Stork, D.G.: Pattern Classification, Ch.9: 483–486 (Wiley, 2001).

[31] Quesada JA, Lopez-Pineda A, Gil-Guillén VF, Durazo-Arvizu R, Orozco-Beltrán D, López-Domenech A, Carratalá-Munuera C (2019). Machine learning to predict cardiovascular risk. Int. J. Clin. Pract..

[32] Rigdon J, Basu S (2019). Machine learning with sparse nutrition data to improve cardiovascular mortality risk prediction in the USA using nationally randomly sampled data. BMJ Open..

[33] Piccialli F, Cuomo S, Crisci D (2020). A deep learning approach for facility patient attendance prediction based on medical booking data. Sci. Rep..

[34] Porumb M, Stranges S, Pescapè A (2020). Precision medicine and artificial intelligence: A pilot study on deep learning for hypoglycemic events detection based on ECG. Sci. Rep..

[35] Vécsei A., Fuhrmann T., Uhl A. Towards automated diagnosis of celiac disease by computer-assisted classification of duodenal imagery. In *4th IET International Conference on Advances in Medical, Signal and Information Processing (MEDSIP 2008), IET*10.1049/cp:20080465 (2008).

[36] Hegenbart S, Uhl A (2015). Vécsei a review survey on computer aided decision support for diagnosis of celiac disease. Comput. Biol. Med..

